# Evaluation of the Physiological Host Range for the Parasitoid *Ooencyrtus mirus*, a Potential Biocontrol Agent of *Bagrada hilaris*

**DOI:** 10.3390/insects11070432

**Published:** 2020-07-10

**Authors:** Nancy Power, Fatemeh Ganjisaffar, Thomas M. Perring

**Affiliations:** Department of Entomology, University of California, 900 University Ave., Riverside, CA 92521, USA; nancy.power@email.ucr.edu (N.P.); thomas.perring@ucr.edu (T.M.P.)

**Keywords:** pentatomidae, encyrtidae, host acceptance, host suitability, bagrada bug, egg parasitoid

## Abstract

The thelytokous egg parasitoid *Ooencyrtus mirus* Triapitsyn and Power (Hymenoptera: Encyrtidae) was recovered from brassica plant debris in Pakistan in an effort to find a biological control agent of the invasive bug *Bagrada hilaris* (Burmeister) (Heteroptera: Pentatomidae) in North America. As the first step in determining the overall host range of this parasitoid, adult females were exposed to the eggs of eight alternate pentatomid host species, two non-pentatomid heteropterans, and two lepidopterans, in choice and no-choice tests. Although *O. mirus* was more successful on *B. hilaris* than the other species in terms of the number of the eggs laid, the number of emerged progeny, and the developmental time of the progeny, it was able to reproduce on all of the alternate hosts except for one of the lepidopterans, whose eggs appeared too small for this parasitoid. The results show *O. mirus* to be a generalist parasitoid species with a preference for *B. hilaris*. The results also indicate that there is a linear relationship between the mean body length of *O. mirus* females and the mean host egg weight with an adjusted $R^{2}$ of 0.90. The implications of this study on the release of *O. mirus* for the control of *B. hilaris* are discussed.

## 1. Introduction

The painted bug, also known as bagrada bug, *Bagrada hilaris* (Burmeister) (Hemiptera: Heteroptera: Pentatomidae) is a serious pest on brassica crops [1]. In its native range of Africa, Asia and the Middle East [2,3], it is one of the major pests of leaf mustard (*Brassica juncea* (L.)) and oilseed brassica crops such as rapeseed and canola (*Brassica napus* L.) [4,5,6]. *Bagrada hilaris* invaded southern Europe in 1978 [7], and North America in 2008, when it first appeared in the United States in Los Angeles County, California [8]. By 2015, it had spread to 24 other counties as well as four contiguous states plus Hawaii [1,9,10,11,12,13] and southward into six states of Mexico [14,15]. In 2016, it appeared in Chile [16]. Active mainly in the warm season, *B. hilaris* nonetheless infests cool-season cole crops by attacking the seedlings in early fall after living on wild mustard weeds in the summer. The feeding damage reduces photosynthesis [17], and often kills the seedlings, reaching up to 60% mortality [1] and leading to incomplete stands of the crop [9,18].

Current management of *B. hilaris* integrates multiple strategies that may include transplanting instead of direct seeding, altering planting dates, destroying crop residues, and controlling mustard weeds in the summer [18]. Other recommended techniques include cultivating the soil frequently during the growing season to destroy *B. hilaris* eggs, reducing nitrogen fertilization, vacuuming the bugs, excluding them by growing the crops under floating row covers, and employing brassica trap crops [6,18]. The principal control method, however, is by application of pyrethroid and neonicotinoid insecticides [19]. Frequent application of insecticides increases the risk of pesticide resistance, as it applies more selection pressure on the pest population [20]. Insecticides also can deplete natural enemy populations, increasing the likelihood of an outbreak of the target or secondary pests. Worker safety, environmental concerns and harm to honeybees [8], along with the cost of using insecticides and the economic importance of brassica crops, are further factors that instigated an effort to expand integrated pest management (IPM) options for *B. hilaris*.

For this purpose, a search for exotic natural enemies as classical biological control agents for *B. hilaris* was initiated in 2014 in Pakistan, where *B. hilaris* is only an occasional pest, likely due to the presence of co-evolved natural enemies. Walker Jones (Research Entomologist, United States Department of Agriculture—Agricultural Research Service (USDA-ARS) Biological Control Laboratory, Stoneville, MS, USA) collaborated with three Pakistani entomologists to send parasitoids obtained from *B. hilaris* eggs in Pakistan to the United States. *Bagrada hilaris* lays eggs individually or in very small clusters of 2–3 eggs on plants or in the soil [1,21]. Therefore, plant debris of leaf mustard and canola in the field [22] were searched, through which three hymenopteran egg parasitoid species were recovered and sent from Pakistan to Jones’ laboratory [23,24]. One of these, a species with thelytokous parthenogenesis recently described as *Ooencyrtus mirus* Triapitsyn and Power (Hymenoptera: Encyrtidae) [25], was sent to the University of California, Riverside (UCR) to be evaluated as a potential biological control agent.

For an exotic species to be released in the field in the United States, a permit must be obtained from the USDA Animal and Plant Health Inspection Service (APHIS). The agency considers host specificity as a major factor in deciding whether to allow the release of a potential biocontrol agent. To that end, the objective of the current study was to determine the host specificity of *O. mirus* by exposing adult females to the eggs of *B. hilaris* and alternate host species.

The first step in determining the overall host range of a potential control agent is to understand the physiological host range; i.e., species on which the parasitoid can survive and develop successfully under controlled conditions in the laboratory [26,27]. This may be followed by behavioral and ecological host range studies to determine which of the physiological hosts the parasitoid may choose and succeed on in the field [27]. The current study was conducted to determine the physiological host range as well as whether *O. mirus* chooses *B. hilaris* over other host species.

## 2. Materials and Methods

### 2.1. Host Sources and Rearing

*Bagrada hilaris* was collected in Riverside, California, and reared in tent-style insect cages (BugDorm^®^-2120, MegaView Science Co., Taiwan) inside two greenhouses at $30\pm 5{\;}^{\circ }$C with ambient humidity and light. They were fed with broccoli (*Brassica oleracea* L. variety Italica), canola (*Brassica napus* L.) and mizuna (*Brassica rapa* L. variety Japonica) seedlings grown in 10 × 10 cm square plastic pots. Adults for experiments were transferred to an insectary room set at $30\pm 1{\;}^{\circ }$C, 40–50% RH, and 14 L:10 D. We placed 15 mating pairs into each of 8–10 round plastic containers (15 cm diameter × 6 cm height) (Durphy^®^ Packaging Co., Warminster, PA, USA), with two 2.5-cm screened holes opposite each other in the sides for ventilation. A piece of white paper towel was cut and placed in the bottom of each container. Daily, the adults were transferred to new containers and supplied with fresh organic broccoli florets. *Bagrada hilaris* eggs for experiments were removed from the containers with a fine brush and gently rubbed off the paper towel by hand.

The alternate hosts studied included eight pentatomid species: the invasive pests *Halyomorpha halys* Stål, *Murgantia histrionica* (Hahn), and *Nezara viridula* (L.), the native pests *Chlorochroa uhleri* (Stål), *Euschistus conspersus* Uhler, *Euschistus servus* (Say), and *Thyanta pallidovirens* (Stål), and the native, beneficial predator *Podisus maculiventris* (Say). Two other heteropterans, *Jadera haematoloma* (Herrich-Schäffer) (Rhopalidae) and *Anasa tristis* (DeGeer) (Coreidae), and two lepidopteran species, *Helicoverpa zea* (Boddie) (Noctuidae) and *Ectomyelois ceratoniae* (Zeller) (Pyralidae) also were tested. These species live in the geographic region where *O. mirus* would be released as a biocontrol agent. All of these species except *J. haematoloma* and *P. maculiventris* are agricultural pests. *Podisus maculiventris* was chosen because it is a beneficial species that preys on other insects. *Jadera haematoloma* was chosen as a representative native, non-pest species. Unlike all the other species in this study, no parasitoids have been recorded at any stage of the *J. haematoloma* life cycle according to Carroll [28] and our own literature search.

*Anasa tristis* was collected in squash fields grown at the Agricultural Operations Facility (Ag Ops) at UCR. *Chlorochroa uhleri*, *E. servus*, *N. viridula*, and *T. pallidovirens* were collected from an alfalfa field at Ag Ops. *Murgantia histrionica* was collected from leaves of bladderpod bushes, *Peritoma arborea* (Nutt.) (Cleomaceae), in the UCR Botanical Gardens. Once established in the insectary, the colonies of these host species were occasionally refreshed with wild individuals. *Jadera haematoloma* was collected under trees on the UCR campus and under bladderpod bushes in the UCR Botanical Gardens. Enough *J. haematoloma* eggs were obtained from newly collected individuals from the field that no offspring were reared past the egg stage. *Ectomyelois ceratoniae* were obtained from dates in the Coachella Valley in Riverside County, California. The *E. conspersus* colony was started with eggs obtained from the USDA-ARS laboratory in Albany, California, and *E. servus* was found in a park near UCR. *Halyomorpha halys* and *H. zea* were obtained from other laboratories in the UCR Entomology Department. *Podisus maculiventris* eggs were purchased from Entomology Solutions, LLC, Louisville, KY, USA, arriving the day after they were laid and used immediately for the host tests.

Most of the alternate host species were reared in the same insectary room as the *B. hilaris* adults, in the same size containers, but with an additional screened hole in the lid for extra ventilation. In addition to the white paper towel in the bottom, the alternate host adult containers had half of a folded brown paper towel laid on top of the food to absorb waste and provide hiding places and an ovipositional substrate. *Anasa tristis* was reared in the laboratory at $23{\;}^{\circ }$C instead of in the insectary. For all the alternate host species, the containers were checked daily; new eggs were collected and fresh food was supplied as needed. The adult bugs were transferred weekly to clean containers with new paper towel. The diet for each alternate host species is listed in Table 1.

### 2.2. Parasitoid Rearing

To provide ovipositional hosts for *O. mirus*, approximately 40 *B. hilaris* eggs were glued (Elmer’s^®^ Products, Inc., Columbus, OH, USA) onto a 1.5 × 4 cm piece of card stock; these egg cards were provided to the parasitoids each day. The eggs were placed in a 9.4 cm height × 2.2 cm diameter glass vial with 10 or 11 3-day-old naïve adult females of *O. mirus* and a cotton plug was inserted in the open end. After 24 h, the wasps were aspirated out, and the vial of exposed eggs was placed on a ridged tray and kept at room temperature (22–$23{\;}^{\circ }$C) under natural light until the new *O. mirus* adults emerged. Each day, newly-emerged wasps were aspirated into a glass vial streaked with honey and placed in a Percival growth chamber (model I30BLL, Perry, IA, USA) at $26\pm 1{\;}^{\circ }$C, 50% RH and 14 L: 10 D until they were ready to be used for the next generation or for the tests at 3 days of age.

### 2.3. Experimental Procedure

*Ooencyrtus mirus* egg parasitism was compared between *B. hilaris* and each of the alternate hosts in choice and no-choice tests, except for *E. ceratoniae*. Since *O. mirus* did not oviposit on *E. ceratoniae* eggs in no-choice tests, we did not proceed with the choice tests for this species. Each choice test replicate consisted of two cards (1.5 × 4 cm); on one of the cards, 10 *B. hilaris* eggs (≤24 h old) were glued in random locations, and on the other card, a cluster of 10 eggs (≤24 h old) of the alternate host species was glued. The placement of eggs mimicked how the eggs are laid by each species. For the pentatomid alternate hosts, rows of eggs were removed from the original cluster and glued so that all eggs within each row were touching other eggs. For the other two heteropterans, the eggs were near each other but not every egg was touching other eggs. For *H. zea*, the eggs were left on the paper towel on which they were laid. A line was drawn with pencil around a group of 10 eggs, and the towel was cut along the line. Usually the eggs were near but not touching each other. The *B. hilaris* card and the alternate host card were placed back-to-back in a 9 cm height × 1 cm diameter glass vial and a cotton plug was inserted in the open end. A 3-day-old naïve *O. mirus* adult female was added to the vial. The vials were placed horizontally, with the cards standing on their long edge, on ridged trays in the same $26{\;}^{\circ }$C growth chamber mentioned above, with 50% RH and 14 L: 10 D. After 24 h, the wasp was removed from the vial with an aspirator. One of the cards was moved to a separate vial to observe the parasitoid emergence for each species of eggs daily.

At the same time the choice tests were being conducted, we also evaluated parasitism in no-choice tests. The no-choice test was similar to the choice test except that each card of 10 eggs was placed in a separate vial from the beginning, and one 3-day-old naïve *O. mirus* adult female was added to each vial for 24 h. The vials were kept in the $26{\;}^{\circ }$C growth chamber. For *E. ceratoniae* the no-choice tests were conducted by cutting a piece of paper towel on which various numbers of eggs were deposited, and exposing the eggs to a 3-day-old *O. mirus* in a 9 × 1 cm glass vial for 24 h. These vials were held at 22–$23{\;}^{\circ }$C under natural light and checked daily.

Parasitism in the choice and no-choice tests was measured in a variety of ways. First, we determined if *O. mirus* oviposited on the host eggs by counting the number of egg pedicels (representative of the parasitoid eggs) under a stereomicroscope. Typical of encyrtids, each *O. mirus* egg has a pedicel that protrudes from the host egg, serving as a respiratory tube for the developing larva [29]. The pedicels were counted between 6 and 12 days after oviposition, after any surviving *B. hilaris* nymphs emerged but before any wasps emerged. For *P. maculiventris*, the pedicels were counted within a few days, because as they mature, the *P. maculiventris* eggs develop long aero-micropylar processes that hide the pedicels. Pedicel counts allowed us to determine whether differences in progeny emergence between *B. hilaris* and the other hosts was due to *O. mirus* laying fewer eggs, lower survival of the eggs, or both. We also determined the level of *O. mirus* superparasitism (parasitizing a host egg more than once by a single parasitoid species indicated by more than one pedicel per egg) on the different host eggs.

Second, all parasitoid-exposed host eggs were checked daily for emergence for 28 days following oviposition. Any host immatures that emerged were removed to prevent them from cannibalizing the remaining eggs. For each emerged parasitoid, the number of days from oviposition to emergence (developmental time) was recorded. Emerged wasps were removed, sexed, and stored in microcentrifuge tubes of ethanol for measuring their body length.

Third, body lengths of up to 20 *O. mirus* adults emerged from each host species were measured from the anterior end of the head to the posterior end, not including the ovipositor, under a Leica Wild M10 stereoscope (Cambridge Scientific Products, MA, USA). A Bausch and Lomb^®^ micrometer with 0.1 mm and 0.01 mm scales was used to calibrate a ruler in one of the eyepiece lenses of the microscope. In addition, we weighed 3–10 egg clusters of *B. hilaris*, *T. pallidovirens*, *N. viridula*, *E. conspersus*, *E. servus*, *M. histroinica*, *C. uhleri*, and *A. tristis* to determine if parasitoid body size was related to host egg size. The eggs were weighed as clusters to avoid damaging eggs, after which the weight was divided by the number of eggs in the cluster to get an estimate of each egg weight.

Finally, we evaluated the reproductive success of parasitoids that emerged from eggs of five of the alternate host species that we were still testing. Five wasps, each from *E. conspersus*, *E. servus*, *P. maculiventris*, *J. haematoloma*, and *H. zea* eggs, were placed into separate glass vials streaked with honey and plugged with cotton. After 3 days, a card of five *B. hilaris* eggs was added to each vial for 24 h to see if the wasps could reproduce on *B. hilaris* after being reared on the alternate host. These parasitoid-exposed eggs were checked daily and the number of emerged progeny was recorded.

### 2.4. Statistical Analysis

All data were analyzed in R [30], and were evaluated for significance at *p* < 0.05. The number of pedicels per 10 host eggs and the number of wasps emerged from those eggs (discrete data) were compared between *B. hilaris* and the alternate hosts using the nonparametric Wilcoxon signed rank test for the paired choice data and the Wilcoxon-Mann-Whitney test for the no-choice data. Parasitoid egg survival, first day of emergence, and developmental time (continuous data) were tested for normality using Shapiro-Wilk test (sample size < 50) or Jarque-Bera test (sample size > 50) at *p* < 0.05. The data did not have a normal distribution, therefore the Wilcoxon-Mann-Whitney test was used for comparing means in each group. For all hypothesis tests, the reported *p* values were from the two-sided tests. Both host egg weight and *O. mirus* body lengths were compared between different hosts using one-way ANOVA followed by the nonparametric Dunn’s Kruskal–Wallis multiple comparison. The linear relationship between the mean host egg weight and the mean *O. mirus* body length was also determined in R.

## 3. Results

### 3.1. Number of *O. mirus* Eggs (Pedicels) Per Host Egg

In the choice tests, the mean number of *O. mirus* eggs (as determined by the number of pedicels) laid within 24 h was significantly lower for *C. uhleri*, *E. conspersus*, *E. servus*, *M. histrionica*, *H. halys*, *A. tristis*, and *H. zea* than for *B. hilaris* (*p* ≤0.046) (Table 2). Among these non-preferred hosts, the mean number of pedicels on *B. hilaris* eggs ranged from 4.9 ± 0.9 in the *B. hilaris*–*A. tristis* pair to 7.6 ± 0.6 in the *B. hilaris*–*H. zea* pair. The mean number of pedicels on the alternate host species in this group ranged from 0.3 ± 0.1 in *B. hilaris*–*C. uhleri* pair to 1.1 ± 0.4 in *B. hilaris*–*M. histrionica* pair. In pairs for which their mean number of pedicels were not significantly different (*p* > 0.05), 3.7 to 3.9 and 1.5 to 3.3 eggs were recorded on *B. hilaris* and alternate host eggs, respectively (Table 2).

In the no-choice tests, the number of parasitoid eggs laid in 24 h was significantly lower in *C. uhleri*, *H. halys*, *M. histrionica*, *P. maculiventris*, *A. tristis*, *J. haematoloma*, and *H. zea* (*p* ≤0.003) (Table 2). Among these host species, the mean number of pedicels on *B. hilaris* eggs ranged from 6.2 ± 0.6 in *B. hilaris*–*C. uhleri* pair to 7.8 ± 0.6 in *B. hilaris*–*P. maculiventris* pair. The mean number of pedicels on the alternate host species in this group ranged from 1.2 ± 0.4 in *B. hilaris*–*H. zea* pair to 5.0 ± 0.7 in *B. hilaris*–*J. haematoloma* pair. In pairs for which their mean number of pedicels were not significantly different, 6.5 to 7.4 and 5.1 to 8.2 eggs were recorded on *B. hilaris* and alternate host eggs, respectively (Table 2).

### 3.2. Progeny Emergence

Surviving host nymphs (Heteroptera) or larvae (Lepidoptera) emerged between days 2–12, only from non-parasitized eggs (those with no pedicels). In choice tests, successful emergence of *O. mirus* progeny was significantly higer in *B. hilaris* than in *C. uhleri*, *E. conspersus*, *E. servus*, *H. halys*, *M. histrionica*, *N. viridula*, *T. pallidovirens*, *A. tristis*, and *H. zea* (*p* ≤0.043) (Table 3). Among these species, the mean number of progeny emerged from *B. hilaris* eggs ranged from 3.5 in *B. hilaris*–*H. halys* and *B. hilaris*–*N. viridula* pairs to 6.9 in *B. hilaris*–*H. zea* pair. The number of emerged progeny did not differ significantly between *B. hilaris* (3.7 wasps) and *P. maculiventris* (2.7 wasps) (*p* = 0.408), or between *B. hilaris* (3.4 wasps) and *J. haematoloma* (1.5 wasps) (*p* = 0.111).

In no-choice tests, emergence of *O. mirus* progeny was significantly higher for *B. hilaris* than for *C. uhleri*, *H. halys*, *M. histrionica*, *P. maculiventris*, *A. tristis*, *J. haematoloma*, and *H. zea* (*p* ≤0.001). Among these species, the mean number of progeny emerged from *B. hilaris* eggs ranged from 5.5 in *B. hilaris*–*C. uhleri* pairs to 7.4 in *B. hilaris*–*H. halys* pairs. Emergence from *B. hilaris* eggs did not differ significantly from *E. conspersus* (*p* = 0.251), *E. servus* (*p* = 0.325), *N. viridula* (0.080), or *T. pallidovirens* (*p* = 0.987) (Table 3). The variances were homogeneous between *B. hilaris* and each alternate host, indicating that the data had similar distributions.

### 3.3. Survival (Proportion of O. mirus Progeny from Parasitized Host Eggs)

The proportion of successful emergence of *O. mirus* progeny from *B. hilaris* eggs ranged from 0.83 to 0.99 in the choice tests, and from 0.92 to 1.00 in the no-choice tests. For the alternate hosts, the emergence ranged from 0.73 to 1.33 in the choice tests, and from 0.77 to 1.70 in the no-choice tests (Table 4). For most of the alternate host species evaluated, the proportion of successful emergence from parasitized host eggs (those with one or more pedicels) did not differ significantly between *B. hilaris* and the alternate host in either the choice or no-choice tests (*p* > 0.05). This was true even for *H. zea* eggs, which were much smaller than all the other host eggs. One exception was that parasitoid emergence was significantly higher from *B. hilaris* (0.99 ± 0.01) than from *J. haematoloma* eggs (0.79 ± 0.07) in both the choice and no-choice tests (*p* < 0.05). In contrast, progeny emergence per parasitized host egg was lower in *B. hilaris* (0.97 ± 0.02) than in *H. halys* (1.70 ± 0.31) in the no-choice test (*p* < 0.01) (Table 4). This was due to superparasitism and the fact that more wasps emerged per host egg in *H. halys*. Because of the more successful superparasitism in *H. halys*, we compared the per pedicel emergence in addition to the per parasitized egg emergence for this host species. For the choice tests, the mean proportions of the per pedicel emergence were 0.88 ± 0.12 (N = 9) and 0.64 ± 0.18 (N = 7) from *B. hilaris* and *H. halys* eggs, respectively, which were not significantly different (*p* = 0.17). For the no-choice tests, the means were 0.96 ± 0.02 (N = 15) and 0.73 ± 0.11 (N = 10), respectively, which also did not differ significantly (*p* = 0.12).

### 3.4. First Day of Emergence and Developmental Time

**Figure 1:**
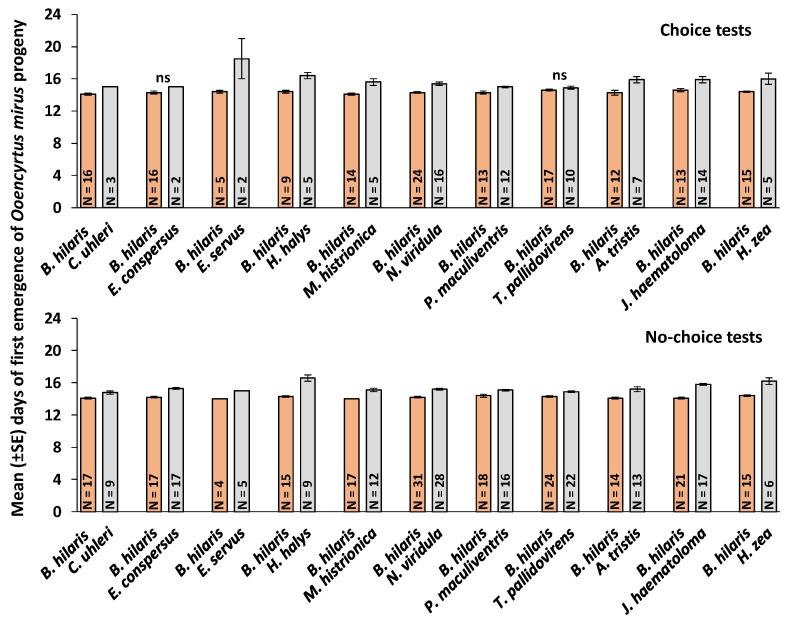
Mean (±SE) days of first emergence of *Ooencyrtus mirus* adults from *Bagrada hilaris* versus alternate hosts in choice and no-choice tests. N is the number of replicates. Means for the pairs with “ns” (non-significant) did not differ significantly (Wilcoxon–Mann–Whitney test, *p* > 0.05).

In the choice test, the mean first day of parasitoid emergence was significantly earlier from *B. hilaris* than from the alternate hosts (*p* < 0.05) except for *E. conspersus* (*p* = 0.098) and *T. pallidovirens* (*p* = 0.362). In the no-choice tests, the mean first day of emergence was significantly earlier from *B. hilaris* than from all the alternate hosts (*p* < 0.01) (Figure 1).

The mean developmental time (egg to progeny emergence) ranged from 14.3–15.0 days on *B. hilaris*, and from 14.9–18.5 days on the alternate hosts in the choice and no-choice tests (Table 5). Most of the *O. mirus* immatures completed their development in 14–16 days, but the developmental time ranged from 13–24 days, with only one wasp emerging on days 13 and 24. For all of the alternate host species in both choice and no-choice tests, the mean developmental time at $26{\;}^{\circ }$C was shorter in *B. hilaris* than in the alternate host (*p* ≤0.02) (Table 5).

Regarding sex ratio of the emerged adults, since *O. mirus* reproduces by thelytokous parthenogenesis, progeny from all host species were females, except four males that emerged from *E. conspersus* eggs.

### 3.5. Host Egg Weight and *O. mirus* Body Length

**Figure 2:**
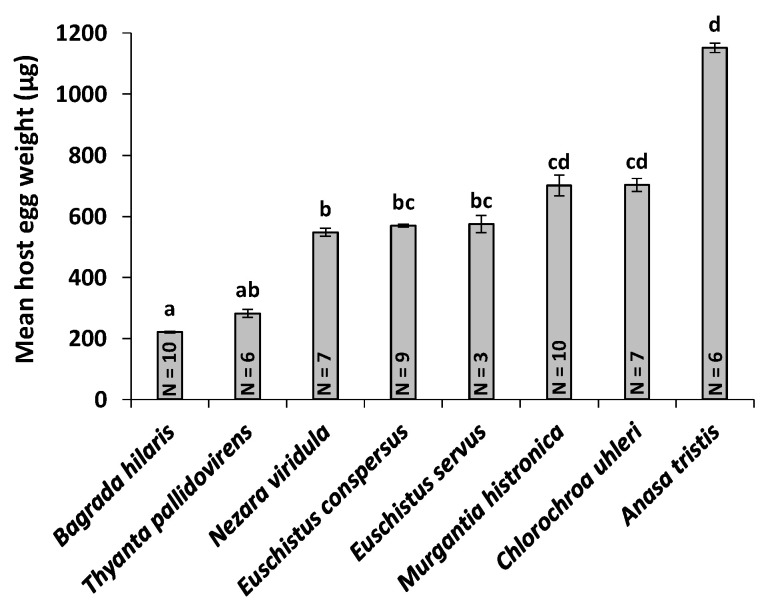
Comparison of mean (±SE) egg weight among different hosts. N is the number of replicates. Significant differences are represented by different letters (Dunn’s Kruskal–Wallis multiple comparison, *p* < 0.05).

**Figure 3:**
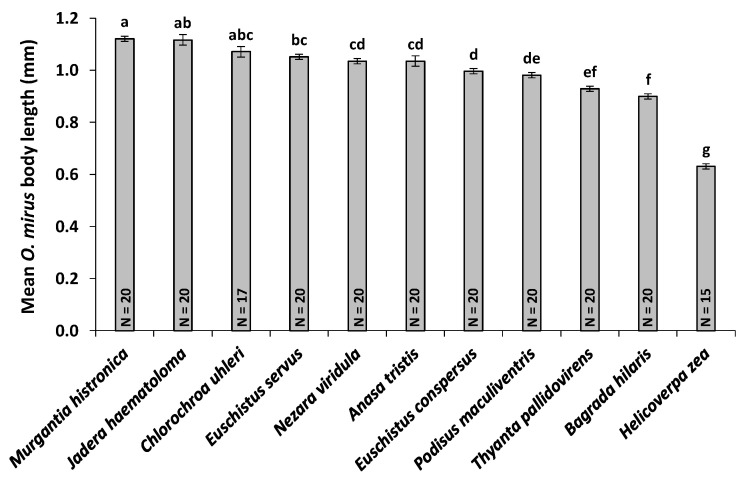
Comparison of mean (±SE) body length of *Ooencyrtus mirus* reared on the respective host eggs. N is the number of replicates. Significant differences are represented by different letters (Dunn’s Kruskal–Wallis multiple comparison, *p* < 0.05).

**Figure 4:**
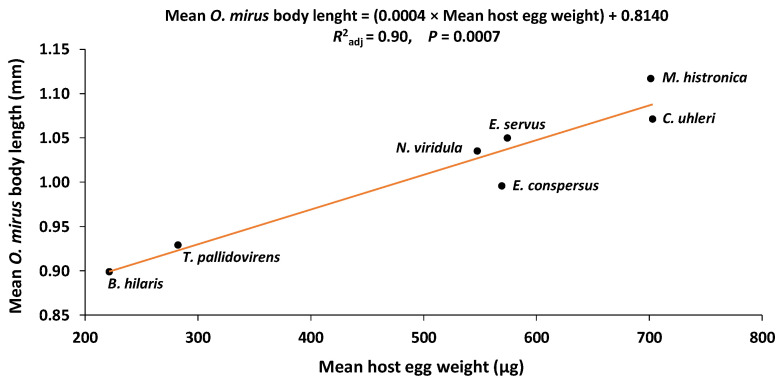
Linear relationship between mean *Ooencyrtus mirus* body length and the respective mean host egg weight.

Egg weights (an indication of size) of the various stink bug hosts differed significantly (*F*${}_{7,\;50}$ = 199.1, *p* < 0.001). *Bagrada hilaris* eggs weighed significantly less than those of all the alternate host species whose eggs were weighed (*N. viridula*, *E. conspersus*, *E. servus*, *M. histrionica*, *C. uhleri*, and *A. tristis*), except *T. pallidovirens* (*p* < 0.05) (Figure 2). Adult *O. mirus* females emerged from different host species also differed significantly in body length (*F*${}_{10,\;198}$ = 80.21, *p* < 0.001). The body length of *O. mirus* females that emerged from *B. hilaris* eggs (0.90 ± 0.01 mm) was significantly larger than those from *H. zea* eggs (0.63 ± 0.01 mm), but smaller than all other host species (0.98–1.12 mm), except *T. pallidovirens* (0.93 ± 0.01 mm) (*p* < 0.05) (Figure 3). A linear regression analysis of the mean egg weight per host species versus *O. mirus* adult female body length produced the following regression equation with an adjusted $R^{2}$ of 0.90: Mean *O. mirus* adult female body length = (0.0004 × Mean host egg weight) + 0.8140 (*p* = 0.0007) (Figure 4).

### 3.6. Reproductive Success of *O. mirus* Emerged from the Alternate Host Eggs

The five *O. mirus* adult females that emerged from the eggs of five alternate host species and subsequently exposed to five *B. hilaris* eggs in separate vials for 24 h, successfully produced F${}_{2}$ progeny on those eggs. The mean number of progeny that emerged from *E. servus* and *J. haematoloma* eggs was 5.0 ± 0.0 (100% emergence rate). An average of 4.4 ± 0.2 and 4.4 ± 0.4 progeny emerged from wasps reared on *P. maculiventris* and *E. conspersus* eggs, respectively (88% emergence rate). The mean number of progeny that emerged from *H. zea* eggs was 1.6 ± 0.4 (32% emergence rate).

## 4. Discussion

The physiological host range tests indicate that *O. mirus* is a generalist with a broad host range but it has an innate host preference for, and greater reproductive success on, *B. hilaris*. It appears that *T. pallidovirens* and *N. viridula* are the most suitable hosts for *O. mirus* after *B. hilaris*. The number of pedicels laid on these species did not differ from *B. hilaris* regardless of whether the parasitoids had a choice or not. Progeny emergence was significantly higher in *B. hilaris* in the choice tests, but it was the same as these alternate hosts in the no-choice tests. Since survival per parasitized egg in *T. pallidovirens* and *N. viridula* was the same as for *B. hilaris* in both choice and no-choice tests, lower emergence from these host species in the choice tests was due to fewer pedicels being laid by *O. mirus* (i.e., lower host acceptance) rather than to lower survival per parasitized alternate host egg (i.e., lower host suitability). *Nezara viridula* is native to Africa [31] but has dispersed to Asia and the Mediterranean region [32]. Therefore *O. mirus* preference for this host species is reasonable. However, *O. mirus* has never existed in sympatry with *T. pallidovirens*, a species native to North America [33]. Our assumption is that since *T. pallidovirens* was the only host whose egg weight was not significantly different from that of *B. hilaris*, the egg size has an impact on the *O. mirus* host selection even though the parasitoids have never been exposed to such host eggs in their native range. It has been shown that physical characteristics of the eggs, such as size, shape, and color can affect the host preference of parasitoid wasps [34,35,36,37].

Given a choice between *B. hilaris* and either *Euschistus* species, *O. mirus* laid more eggs on *B. hilaris*, but in no-choice tests they laid a similar number of eggs on the *Euschistus* spp. eggs as on *B. hilaris* eggs. Thus *O. mirus* prefers *B. hilaris* to *Euschistus* spp., but will accept them as a host in the absence of *B. hilaris*.

When given a choice between *B. hilaris* and *P. maculiventris* or *B. hilaris* and *J. haematoloma*, *O. mirus* females laid a similar number of eggs as on *B. hilaris*. Given no choice, however, they laid significantly fewer eggs on the alternate host than on *B. hilaris*. Perhaps some factor in the *B. hilaris* eggs stimulated the wasps to oviposit and once stimulated in the choice test, the parasitoids oviposited regardless of host species. Since *B. hilaris* was the parasitoid rearing host, it is reasonable that they may be stimulated to oviposit in the presence of *B. hilaris* eggs. This hypothesis needs to be tested through chemical ecology studies involving chemical components of the host eggs and behavioral observations. In addition, to the best of our knowledge, *O. mirus* is the first parasitoid that has been able to reproduce on *J. haematoloma*, and the fact that this was the only host tested on which the survival was lower than on *B. hilaris* suggests that *J. haematoloma* eggs might have an ability to suppress the development of the parasitoids.

*Ooencyrtus mirus* laid significantly more eggs in *B. hilaris* than in *C. uhleri*, *H. halys*, *M. histrionica*, *A. tristis*, and *H. zea* in both the choice and no-choice tests. Likewise, significantly more wasps emerged from *B. hilaris* eggs than from these alternate host eggs in both the choice and no-choice tests. These alternate species are thus the least preferred hosts for *O. mirus*. One of the species in this group, *H. halys*, had the largest eggs evaluated. While *O. mirus* adult females typically laid only one egg per host egg on the other hosts, they laid 2–3 eggs per *H. halys* egg, with the highest incidence of superparasitism of all the alternate hosts. In addition, on most of the other hosts, the infrequent number of eggs with two pedicels usually produced only one *O. mirus* adult, but on *H. halys*, superparasitized eggs typically produced 2–3 *O. mirus* adults. When only one or two wasps emerged from a *H. halys* egg, the wasps were visibly larger than normal, but when three wasps emerged, they looked similar in size to those from other alternate host eggs (except for *H. zea*, which produced the smallest wasps). The fact that *O. mirus* superparasitized the highest percentage of eggs on *H. halys*, the largest eggs, and none on *H. zea*, the smallest eggs, along with intermediate percentages on the intermediate size eggs, also supports the idea that female *O. mirus* adults can discern the host egg size and choose the number of eggs to lay on a given host egg based in part on host egg size. *Ooencyrtus mirus* thus can be considered a gregarious parasitoid on *H. halys*, whereas it is mostly a solitary parasitoid on the other species tested.

Besides the species mentioned, *O. mirus* could also parasitize the pentatomid *Chlorochroa ligata* (Say); however, eggs of this species were available for only one replication of each test. In the choice test, the *C. ligata* egg card had three parasitized eggs, from which a single *O. mirus* progeny emerged, and in the no-choice test, the *C. ligata* egg card had six parasitized eggs, from which five wasps emerged. The host range of *O. mirus* might thus be broader than the species tested in this study as it is common among egg parasitoids of the Pentatomidae to use multiple stink bug host species, or a combination of pentatomid and lepidopteran hosts. For example, Talamas et al. [38] report *Trissolcus basalis* (Wollaston) emerging from 30 different pentatomid host species, and seven other species of *Trissolcus* each emerging from 2–5 pentatomids. Furthermore, *Trissolcus hullensis* (Harrington) and *Trissolcus thyantae* Ashmead each are known to parasitize two pentatomids and a lepidopteran species [38]. Likewise, the parasitoid *Trissolcus brochymenae* Ashmead (syn. *Trissolcus murgantiae*) is associated with at least 11 pentatomid species occurring in the New World [39]. Samra et al. [40] found that *Ooencyrtus pityocampae* Mercet, an egg parasitoid of *Thaumetopooea wilkinsoni* Tams (Lepidoptera: Notodontidae) can also parasitize the pentatomid *Stenozygum coloratum* (Klug). However, we assume despite its wide host range, the preference of *O. mirus* for *B. hilaris* and greater parasitism and emergence rates on the eggs of this invasive pest would likely reduce parasitism on non-target heteropterans in field. Botch and Delfosse [41] suggested a similar result for the parasitoid *Trissolcus japonicus* (Ashmead) (Hymenoptera: Scelionidae), which has been evaluated for biological control of *H. halys*.

It is important to note that most of the alternate hosts tested in the present study, whether native or not, are agricultural pests themselves, so *O. mirus* potentially could assist in managing multiple pest insects if introduced in North America. However, the fact that it also parasitizes *P. maculiventris*, a beneficial species, could limit its release to the areas where *P. maculiventris* is not an important natural enemy. This assumption, however, requires additional studies to understand the dispersal ability of the parasitoid. The approved release of natural enemies whose physiological host range includes native, non-target hosts has occurred in the past. For example, the USDA APHIS granted permission to release the parthenogenetic egg parasitoid *Oobius agrili* Zhang and Huang (Hymenoptera: Encyrtidae) against the emerald ash borer, *Agrilus planipennis* Fairmaire (Coleoptera: Buprestidae) in Michigan [42]. In that case, no-choice tests had shown that *O. agrili* could attack native *Agrilus* beetle species, but choice tests revealed that the parasitoid greatly preferred to oviposit on the target host, *A. planipennis*, on ash trees (*Fraxinus* spp.) than on non-target hosts on their respective host plants. The same biological control effort also received APHIS approval for release of a braconid larval parasite of *A. planipennis*, *Spathius agrili* Yang. Like *O. agrili*, this species showed a physiological host range overlap with native *Agrilus* species. However, Y-tube olfactometer tests showed that *S. agrili* was only attracted to three host tree species, suggesting that if parasitoids are not attracted to the host trees in the wild, they will be unlikely to encounter and parasitize the non-target non-target hosts. In addition, field-collected larvae of six other *Agrilus* species in the native range of *S. agrili* in China produced no *S. agrili*, suggesting a narrow host range in the field. Furthermore, the success rate was lower in non-target hosts in no-choice tests in the laboratory [42]. Similarly, future choice tests that include the hosts’ respective host plants, olfactometer studies of *O. mirus*’ searching behavior, and investigation of its actual host range in the field in its native region, could help assess the impact *O. mirus* might have on non-target species. Such studies could further inform its suitability as a potential biological control agent for *B. hilaris* in North America.

Although *O. mirus* is a parasitoid with a broad host range, its preference for *B. hilaris* eggs could limit negative effects on non-target hosts. In addition, *O. mirus* has other traits that characterize an effective biocontrol agent. First, no parasitized host eggs survived; i.e., no egg with a pedicel produced a host offspring. Such parasitoid-induced host egg mortality may add to the biological control impact of the parasitoid [43]. It also may signal that oviposition by *O. mirus* is accompanied by venom or substantial physical damage to the host [43,44,45,46,47]. Second, *O. mirus* is parthenogenetic, predominantly producing females that can lay eggs without needing to find a mate. Third, *O. mirus* does not need to diapause, but it can go into an arrested developmental state in the larval stage at $14{\;}^{\circ }$C and $16{\;}^{\circ }$C and then revives in warmer temperatures [48]. This could be used in mass-rearing by cold-storing parasitized host eggs when adults are not needed for release. Fourth, *O. mirus* has a short life cycle, which can be as low as 10 days at $32{\;}^{\circ }$C, the highest constant temperature that still produces mostly females in the F${}_{2}$ generation [48]. Finally, parasitized *B. hilaris* eggs show high survival rates of 80–100% in the lab, and in the 4 years we have had the insect in colony, it has not experienced any diseases, hyperparasitoids or other limits to its population growth. In addition, *O. mirus* can be raised on alternate hosts. We have maintained a colony of *O. mirus* on *T. pallidovirens* and *N. viridula* eggs for several years, and even on the less suitable *C. uhleri* eggs for several months. These colonies survived until no more host eggs were added. These long-term alternate host colonies demonstrate that *O. mirus* can sustain a population without needing *B. hilaris* as even an intermittent host. *Ooencyrtus mirus* might also find and use alternate hosts in the field to maintain its population when *B. hilaris* individuals are scarce. These characteristics encourage further investigation of *O. mirus*’s ability to discriminate between target and non-target hosts.

## 5. Conclusions

This study revealed that *O. mirus* is a generalist species that can parasitize eggs of a wide range of hosts. It has an innate preference for *B. hilaris* eggs and shows greater reproductive success on those eggs. The preference of *O. mirus* for *B. hilaris* eggs could limit negative effects on non-target hosts and could assure its survival in the field when *B. hilaris* populations are low.

## Figures and Tables

**Table 1:** Alternate host diets.

Alternate Host Species	Diet
*Anasa tristis*	Fruits and fresh leaves of squash, *Cucurbita moschata* L. variety Black Futsu
*Chlorochroa uhleri*	Fresh green beans, raw peanuts, raw sunflower seeds, and bouquets of Russian thistle, *Salsola tragus* L., and alfalfa, *Medicago sativa* L.
*Ectomyelois ceratoniae*	Lab-prepared mixture of soy meal, sugar and water
*Euschistus conspersus*	Alfalfa bouquet, fresh green beans, raw peanuts, raw sunflower seeds, raw pistachio nut meats, and broccoli floret
*Euschistus servus*	Alfalfa bouquet, fresh green beans, raw peanuts, and raw sunflower seeds
*Halyomorha halys*	Apples, avocados, carrots, grapes, fresh green beans, and fresh cuttings of empress tree, *Paulownia tomentosa* (Thunb.), and butterfly bush, *Buddleja davidii* Franchet
*Helicoverpa zea*	Lepidoptera Diet—Product “F9772-Tray”, Frontier Agricultural Sciences, Newark, DE, USA
*Jadera haematoloma*	Bouquet of bladderpod, *Peritoma arborea* (Nutt.)
*Murgantia histrionica*	Bouquet of bladderpod, *Peritoma arborea* (Nutt.)
*Nezara viridula*	Fresh green beans, raw peanuts, raw sunflower seeds
*Podisus maculiventris*	N/A
*Thyanta pallidovirens*	Fresh green beans, raw peanuts, raw sunflower seeds, and broccoli floret

Bouquet refers to fresh stems with leaves in 7.5 cm aquatubes with lids cut to hold the stems (Syndicate Sales Inc., Kokomo, IN, USA). The Russian thistle and alfalfa were collected from the UCR campus and Ag Ops, respectively. Sunflower seeds were glued to a 2.5 × 2 cm piece of card stock for easier handling. All diet ingredients were organic.

**Table 2:** Mean (±SE) number of *Ooencyrtus mirus* eggs (pedicels) laid within 24 h on 10 host eggs.

Host Species		Choice			No-Choice		
		N	Mean ± SE	*p*	N	Mean ± SE	*p*
**Pentatomidae**	*B. hilaris*	19	6.5 ± 0.7	<0.001	18	6.2 ± 0.6	<0.001
	*C. uhleri*		0.3 ± 0.1			1.9 ± 0.5	
	*B. hilaris*	17	7.2 ± 0.7	<0.001	17	7.4 ± 0.4	0.294
	*E. conspersus*		0.5 ± 0.4			7.1 ± 0.6	
	*B. hilaris*	5	6.4 ± 1.1	0.021	5	6.6 ± 1.8	0.786
	*E. servus*		0.4 ± 0.2			8.2 ± 0.9	
	*B. hilaris*	15	3.9 ± 1.0	0.046	15	7.6 ± 0.4	<0.001
	*H. halys*		1.5 ± 0.5			2.0 ± 0.5	
	*B. hilaris*	16	5.3 ± 0.8	0.002	16	7.1 ± 0.5	<0.001
	*M. histrionica*		1.1 ± 0.4			2.0 ± 0.6	
	*B. hilaris*	17	3.9 ± 0.7	0.107	17	6.5 ± 0.6	0.069
	*N. viridula*		2.3 ± 0.7			5.1 ± 0.7	
	*B. hilaris*	20	3.9 ± 0.9	0.344	20	7.8 ± 0.6	<0.001
	*P. maculiventris*		3.3 ± 0.7			4.9 ± 0.6	
	*B. hilaris*	13	3.8 ± 1.0	0.089	13	6.8 ± 0.5	0.857
	*T. pallidovirens*		2.2 ± 0.7			7.5 ± 0.4	
**Other Heteroptera**	*B. hilaris*	16	4.9 ± 0.9	0.004	15	7.7 ± 0.4	<0.001
	*A. tristis*		1.0 ± 0.4			2.3 ± 0.4	
	*B. hilaris*	21	3.7 ± 0.8	0.255	21	7.4 ± 0.4	0.003
	*J. haematoloma*		3.3 ± 0.7			5.0 ± 0.7	
**Lepidoptera**	*B. hilaris*	15	7.6 ± 0.6	<0.001	15	7.3 ± 0.5	<0.001
	*H. zea*		0.8 ± 0.4			1.2 ± 0.4	

N is the number of replicates. Data for each group were analyzed using Wilcoxon signed rank test for the choice test, and Wilcoxon–Mann–Whitney test for the no-choice test. The number of pedicels for *C. uhleri* may be underestimated. For this species, the black base of the pedicels, which distinguishes the pedicels from host micropylar processes, was buried in the host chorion and not visible.

**Table 3:** Mean (±SE) number of progeny emerged from 10 host eggs exposed to one *Ooencyrtus mirus* adult female for 24 h.

Host Species		Choice				No-Choice			
		N	# Progeny Emerged	Mean ± SE	*p*	N	# Progeny Emerged	Mean ± SE	*p*
**Pentatomidae**	*B. hilaris*	19	110	5.8 ± 0.7	<0.001	19	104	5.5 ± 0.5	<0.001
	*C. uhleri*		5	0.3 ± 0.1			28	1.5 ± 0.4	
	*B. hilaris*	17	111	6.5 ± 0.7	<0.001	17	108	6.4 ± 0.5	0.251
	*E. conspersus*		6	0.4 ± 0.3			92	5.4 ± 0.5	
	*B. hilaris*	5	29	5.8 ± 1.0	0.043	5	32	6.4 ± 1.7	0.325
	*E. servus*		2	0.4 ± 0.2			28	5.6 ± 0.7	
	*B. hilaris*	15	52	3.5 ± 0.8	0.040	15	111	7.4 ± 0.4	<0.001
	*H. halys*		18	1.2 ± 0.4			24	1.6 ± 0.5	
	*B. hilaris*	17	87	5.1 ± 0.7	0.002	17	115	6.8 ± 0.5	<0.001
	*M. histrionica*		14	0.8 ± 0.4			37	2.2 ± 0.6	
	*B. hilaris*	31	110	3.5 ± 0.5	0.014	31	171	5.5 ± 0.4	0.080
	*N. viridula*		44	1.4 ± 0.3			134	4.3 ± 0.4	
	*B. hilaris*	20	73	3.7 ± 0.7	0.408	20	136	6.8 ± 0.5	<0.001
	*P. maculiventris*		54	2.7 ± 0.6			70	3.5 ± 0.5	
	*B. hilaris*	23	91	4.0 ± 0.7	0.021	24	138	5.8 ± 0.3	0.987
	*T. pallidovirens*		40	1.7 ± 0.5			131	5.5 ± 0.6	
**Other Heteroptera**	*B. hilaris*	16	76	4.8 ± 0.9	0.004	15	105	7.0 ± 0.3	<0.001
	*A. tristis*		13	0.8 ± 0.3			34	2.3 ± 0.4	
	*B. hilaris*	21	72	3.4 ± 0.8	0.111	21	149	7.1 ± 0.4	<0.001
	*J. haematoloma*		32	1.5 ± 0.3			60	2.9 ± 0.5	
**Lepidoptera**	*B. hilaris*	15	104	6.9 ± 0.6	<0.001	15	102	6.8 ± 0.5	<0.001
	*H. zea*		8	0.5 ± 0.2			14	0.9 ± 0.4	

N is the number of replicates. # refers to the numbers of progeny. Data for each group were analyzed using Wilcoxon signed rank test for the choice test, and Wilcoxon–Mann–Whitney test for the no-choice test.

**Table 4:** *Ooencyrtus mirus* immature survival: mean (±SE) proportion of emerged progeny from host eggs with one or more *O. mirus* egg pedicels.

Host Species		Choice			No-Choice		
		N	Mean ± SE	*p*	N	Mean ± SE	*p*
**Pentatomidae**	*B. hilaris*	17	0.98 ± 0.02	0.169	16	0.92 ± 0.03	0.183
	*C. uhleri*	3	1.33 ± 0.33		10	1.03 ± 0.17	
	*B. hilaris*	16	0.98 ± 0.02	0.974	17	0.91 ± 0.03	0.085
	*E. conspersus*	2	1.00 ± 0.00		17	1.01 ± 0.04	
	*B. hilaris*	5	0.94 ± 0.04	0.762	4	1.00 ± 0.00	0.444
	*E. servus*	2	1.00 ± 0.00		5	0.90 ± 0.06	
	*B. hilaris*	9	0.96 ± 0.02	0.475	15	0.97 ± 0.02	0.001
	*H. halys*	7	0.83 ± 0.3		10	1.70 ± 0.31	
	*B. hilaris*	14	0.95 ± 0.03	1.000	17	1.00 ± 0.01	0.470
	*M. histrionica*	8	0.79 ± 0.18		13	1.02 ± 0.02	
	*B. hilaris*	13	0.93 ± 0.03	0.084	17	0.94 ± 0.02	0.388
	*N. viridula*	11	0.73 ± 0.10		16	0.98 ± 0.06	
	*B. hilaris*	13	0.98 ± 0.02	0.225	18	0.96 ± 0.02	0.803
	*P. maculiventris*	13	0.85 ± 0.09		16	0.92 ± 0.05	
	*B. hilaris*	9	0.83 ± 0.11	0.945	13	0.94 ± 0.02	0.852
	*T. pallidovirens*	7	0.80 ± 0.14		13	0.94 ± 0.03	
**Other Heteroptera**	*B. hilaris*	12	0.99 ± 0.01	1.000	16	0.98 ± 0.01	0.069
	*A. tristis*	7	1.00 ± 0.11		14	1.07 ± 0.07	
	*B. hilaris*	13	0.99 ± 0.01	0.047	21	0.96 ± 0.02	0.039
	*J. haematoloma*	14	0.79 ± 0.07		18	0.77 ± 0.08	
**Lepidoptera**	*B. hilaris*	15	0.97 ± 0.02	0.284	15	0.95 ± 0.03	0.772
	*H. zea*	5	0.80 ± 0.12		9	0.83 ± 0.20	

N is the number of replicates. Data for each group were analyzed using Wilcoxon–Mann–Whitney test for both choice and no-choice tests. Proportions greater than 1.00 are due to superparasitized eggs from which more than one progeny emerged. Note that *C. uhleri*, *E. conspersus* and *E. servus* had ≤ 3 replicates from which adult *O. mirus* emerged in the choice tests.

**Table 5:** Mean (±SE) developmental time (in days) from egg to adult of *Ooencyrtus mirus* in choice and no-choice tests.

Host Species		Choice			No-Choice		
		# Progeny Emerged	Mean ± SE	*p*	# Progeny Emerged	Mean ± SE	*p*
**Pentatomidae**	*B. hilaris*	110	14.3 ± 0.0	<0.001	104	14.3 ± 0.1	<0.001
	*C. uhleri*	5	15.2 ± 0.2		28	14.9 ± 0.1	
	*B. hilaris*	111	14.5 ± 0.1	<0.001	108	14.4 ± 0.1	<0.001
	*E. conspersus*	6	15.5 ± 0.3		92	15.7 ± 0.1	
	*B. hilaris*	29	14.6 ± 0.1	0.004	32	14.4 ± 0.1	<0.001
	*E. servus*	2	18.5 ± 2.5		28	15.5 ± 0.3	
	*B. hilaris*	52	15.0 ± 0.1	<0.001	111	14.5 ± 0.1	<0.001
	*H. halys*	18	16.1 ± 0.1		24	16.0 ± 0.2	
	*B. hilaris*	87	14.3 ± 0.1	<0.001	115	14.3 ± 0.1	<0.001
	*M. histrionica*	8	15.4 ± 0.3		31	15.4 ± 0.1	
	*B. hilaris*	110	14.7 ± 0.1	<0.001	171	14.6 ± 0.1	<0.001
	*N. viridula*	44	15.7 ± 0.2		134	15.4± 0.1	
	*B. hilaris*	73	14.3 ± 0.1	<0.001	136	14.6 ± 0.1	<0.001
	*P. maculiventris*	54	15.3 ± 0.1		70	15.5 ± 0.1	
	*B. hilaris*	91	14.9 ± 0.1	0.020	138	14.9 ± 0.1	<0.001
	*T. pallidovirens*	40	15.1± 0.1		131	15.2 ± 0.1	
**Other Heteroptera**	*B. hilaris*	76	14.4 ± 0.1	<0.001	105	14.3 ± 0.0	<0.001
	*A. tristis*	13	15.8 ± 0.3		34	15.2 ± 0.2	
	*B. hilaris*	72	14.4 ± 0.1	<0.001	149	14.4 ± 0.1	<0.001
	*J. haematoloma*	32	16.2 ± 0.1		60	16.2 ± 0.1	
**Lepidoptera**	*B. hilaris*	104	14.9 ± 0.1	0.001	102	14.8 ± 0.1	<0.001
	*H. zea*	8	16.1 ± 0.4		14	16.6 ± 0.3	

# refers to the numbers of progeny. Data for each group were analyzed using Wilcoxon-Mann-Whitney test for both choice and no-choice tests.
